# Innovations Lead to Change and State of the Art is Correspondingly Short-Lived: Is There Still a Necessity for Balloon Guide Catheters for Mechanical Thrombectomy in Acute Ischemic Stroke Treatment?

**DOI:** 10.1007/s00270-024-03777-y

**Published:** 2024-06-19

**Authors:** Ansgar Berlis

**Affiliations:** Diagnostic and Interventional Neuroradiology, University Hospital Augsburg, Stenglinstr. 2, 86156 Augsburg, Germany

According to the study published by Knapen et al. [1], it is worth considering whether the use of balloon guide catheters (BGC) in endovascular treatment of acute ischemic stroke is still necessary. This single-center retrospective study was conducted in a high-volume center and presents real-world data acquired between September 2020 and February 2023. A total of 428 patients were included, and BGCs were used in 310 patients (72%). However, the balloon was not inflated during thrombectomy in 210 of those patients (68%). In 118 patients, no BGC was used. The patient groups were comparable, and clinical outcome, which is the most important parameter was best in the group with guide catheters without balloons, especially when direct aspiration was chosen as the first-line strategy. A median of two mechanical thrombectomy maneuvers were needed in this group, while the median number was one in the balloon group. Despite this difference, procedure duration was comparable across all three groups. The complication and mortality rates were lowest in the non-balloon catheter group. These results represent the current state of technical innovation.

Therefore, it is not surprising that the presented data seemingly contradict the findings of the 2021 meta-analysis by Podlasek et al. [2], which included 16 studies with 5507 patients. In that analysis, BGCs had a positive influence on favorable clinical outcomes for both first-line stent retrieval and direct aspiration, but not for the combination of both methods.

The technological advancements in aspiration catheters with larger inner diameters and increased flexibility, which enable access to intracranial vascular occlusions, have significantly improved reperfusion rates. Due to their nearly occlusive external diameters, these catheters can completely remove thrombi, even when used in combination with stent retrievers and direct aspiration. As a result, proximal balloon occlusion for flow reversal is no longer as crucial as it was five years ago, because the occlusive aspiration catheters prevent thrombus fragmentation. This may explain why the balloon was not inflated in 68% of the BGC cases.

In my opinion, BGCs are still indicated when aspiration catheters are not occlusive, particularly for internal carotid artery occlusions. In such cases, flow reversal remains the optimal solution. Hopefully the results randomized ProFATE study, which compares EVT of acute ischemic stroke with and without BGC will shed some more light on this topic [3]. Over the past decade, several paradigms in endovascular stroke treatment have changed or been modified. As a rapidly evolving field, current best practices require frequent and prompt reevaluation.
